# Review and First Study of a Single-Port Robot in Pelvic Traumatology

**DOI:** 10.3390/jcm14196920

**Published:** 2025-09-30

**Authors:** Sebastian Rogenhofer, René Hartensuer

**Affiliations:** 1 Department of Urology and Pediatric Urology, Klinikum Aschaffenburg-Alzenau, Am Hasenkopf 1, D-63739 Aschaffenburg, Germany; sebastian.rogenhofer@klinikum-ab-alz.de; 2 Department of Orthopedics, Traumatology, Hand Surgery and Sports Medicine, Klinikum Aschaffenburg-Alzenau, Am Hasenkopf 1, D-63739 Aschaffenburg, Germany

**Keywords:** robot, pelvis, fracture, single port, da Vinci SP, urethra, trauma

## Abstract

**Background/Objectives**: Robotic procedures are becoming a standard component of modern protocols in some fields of surgery, given that they present many advantages in terms of accuracy among surgical options. However, in the field of pelvic trauma, limited knowledge is available as concerns robot-assisted surgery. **Methods**: In this study, a review of the literature was undertaken, and based on the available knowledge and our own clinical experience, we performed urethral repair and pelvic ring reconstruction using a da Vinci SP robot. **Results**: Given that, to the best of our knowledge, no report on urethral repair in severe trauma via robot-assisted osteosynthesis using a single-port robot has been published thus far, we present a novel study on the use of a single-port robot in pelvic traumatology, including its advantages and limitations. **Conclusions**: Single-port robotic urethral repair in cases of severe pelvic trauma is feasible and presents great advantages, with findings indicating that even single-port robotic-assisted reconstruction of the anterior pelvic ring is possible. However, our study contained limitations, and additional small incisions for reposition and screw placement purposes were necessary during surgery.

## 1. Introduction

Robotic surgery has become a transformative technology in several surgical fields, such as colorectal, gynecological, and urological surgery. With the approval of the da Vinci system (Intuitive Surgical, Sunnyvale, CA, USA) for general surgery in the 1990s, robotic surgery has come to be seen as a breakthrough technology, especially in urology [1]. Furthermore, since the introduction of robotic urology in 2001 by Jochen Binder in Frankfurt [2], there have been remarkable innovations introduced to minimize surgical invasiveness. The latest development in this field is single-port robots (SPs), with one recent study reporting SPs as having satisfactory intraoperative performance compared to the multi-port Xi robot in radical prostatectomy [3]. The da Vinci SP (single port) surgical system offers enhanced precision and control by making a single, small incision, thus minimizing tissue trauma and improving cosmetic outcomes. Its flexible, wristed instruments and 3D high-definition visualization features allow surgeons to perform complex procedures in confined spaces with greater dexterity and accuracy than before [4].

The treatment of complex pelvic trauma remains challenging. Despite advances in trauma treatment, mortality from severe pelvic fractures is reported to be 4.7% [5]. A recent review found that minimally invasive percutaneous techniques result in reduced soft tissue trauma and consecutive shorter recovery times. However, open surgery is still considered to ensure superior anatomical reposition and restitution. The authors of the review conclude that clinicians must evaluate both principles when determining treatment and consider patient-specific factors in order to aim for optimal treatment for an individual [6]. In other words, one must judge between the reduced burden of surgery from minimally invasive procedures and the increased reduction of the pelvic ring from open surgery—factors that will have a relevant impact on the outcome of the treatment, as indicated by Yu et al., who found that functional outcomes were improved based on the quality of reduction of the pelvic ring [7].

Only a few reports can be found on the usage of robot technology in pelvic traumatology [1,8,9,10,11,12], with the available reports restricted to limited use of multi-port robots [8] or the usage of robot-based trajectory navigation and reposition [1,9,11,12]. In general, robot-assisted pelvic surgery is considered to be beneficial in terms of shorter operation times, reduced intraoperative fluoroscopy times, and increased precision compared to conventional surgery. However, the studies reporting these benefits mainly focus on percutaneous screw placement into the pelvis [13]. Alternatively, multi-port robots such as the da Vinci Xi system are reported to be capable of repairing pre-sacral nerves [10], decompressing ulnar nerves, and repairing supraclavicular brachial plexus dissection, with reports of their use in anterior lumbar interbody fusion [14,15]. However, in orthopedics, robot use remains limited.

Seeking the best treatment option for our patient, we performed a comprehensive review of the literature and harnessed the potential advantages of a single-port robot for urethral repair. Additionally, we examined the possibility of using these benefits in pelvic fracture treatment.

As previously mentioned, the use of a single-port robot (da Vinci SP robot) in pelvic traumatology has not been reported thus far. In this study, we performed SP-robot-assisted reconstruction on a type C pelvic fracture combined with traumatic urethra repair and report the findings here for the first time.

## 2. Materials and Methods

Studies in English and German were reviewed using PubMed, Cochrane Central Register, and Google Scholar with the following keywords used: “robot”, “pelvis”, “fracture”, “Da Vinci”, and “traumatology”. All resulting abstracts were screened for the use of robot technology in pelvic trauma. A total of 15 trial studies matched the keywords “robot”, “pelvic”, and “fracture” from a search conducted in the Cochrane Central Register of Controlled Trials. The Google Scholar search, using the terms “robot”, “pelvis”, “fracture”, “Da Vinci”, and “traumatology”, resulted in 34 matches, whereas in PubMed, ((pelvic[MeSH Terms]) AND (robotic[MeSH Terms])) AND (trauma[MeSH Terms]) resulted in 13 reports. Unfortunately, most of these reports were not useful for determining advanced surgery.

After reviewing the available literature, only two reports on robotic surgery of bony pelvic trauma were found [8,16], but neither involved the use of a single-port robot. The limited knowledge found on orthopedic usage was restricted to multi-port robotic systems such as the da Vinci Xi system. Ultimately, the extensive experience in both multi-port and single-port robotic surgery in our robotic center (together with promising reports) guided our decision to utilize the da Vinci SP system in a urological procedure involving minimally invasive repair, as we considered the combination of urethra injury and complex pelvic fracture in our patient to be a compelling opportunity to implement robot-assisted surgery. Thus, due to limited reports on the clinical experience of robot-assisted surgery, we primarily drew on our extensive experience in urological surgery, using the da Vinci Xi and da Vinci SP robots in our robotic center to treat our patient.

### 2.1. Patient Demographics

**Figure 1:**
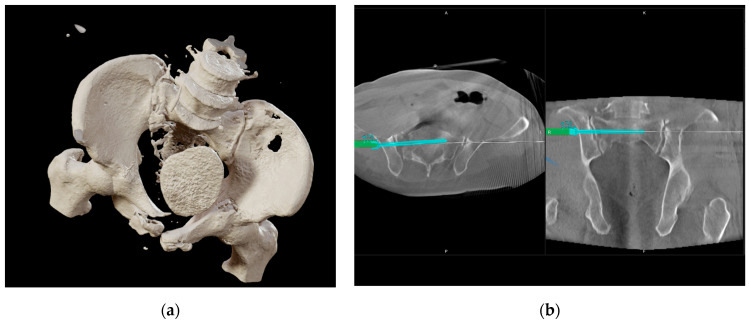
After initial and secondary assessment according to ATLS principles, initial CT diagnostics and primary surgical treatment were performed. (**a**) Initial 3D CT reconstruction of the pelvis, showing the posterior and anterior fracture dislocation in relation to the bladder; (**b**) 3D navigation planning of posterior pelvic ring reconstruction for initial stabilization.

A 54-year-old male patient was admitted to our level 1 trauma center after having been trapped between two tree trunks. With the pelvic binder in place, no acute ABC trauma was obvious. After primary and secondary surveys in accordance with ATLS principles, the patient was found to present a complex pelvic fracture with traumatic urethra disruption (Figure 1a).

### 2.2. Injury Details and Initial Treatment

Initially, 3D navigation (Figure 1b) and external supraacetabular fixation were used to reduce and stabilize the posterior pelvic ring. As transurethral catheter placement was not possible, a suprapubic catheter was placed, with radiological controls demonstrating remaining dislocation in the anterior pelvic straddle fragment.

### 2.3. Indication

Due to the need for urethra reconstruction, we decided to combine early robotic reconstruction with definitive osteosynthesis of the pelvis, based on available research showing the feasibility [8] and first clinical usage [16] of da Vinci Xi robotic surgery in pelvic traumatology. After obtaining informed consent from the patient, with the possibility of conversion to traditional open pelvic trauma surgery at any stage of the procedure, we were able to combine established urological robotic surgery protocols with advanced, minimally invasive trauma care.

A standard setup for robotic prostatectomy and standard pelvic instrumentation was used for the procedure. The patient was placed in the supine position, and an X-ray machine was prepared to facilitate standard intraoperative imaging of the pelvis. However, while the robot was mounted, use of the X-ray machine was limited.

## 3. Results

### 3.1. Literature Review

As previously mentioned, the literature on da Vinci robot usage in pelvic trauma surgery is scarce, with only two reports on da Vinci robotics in orthopedic pelvic trauma available [8,16] and no reports on da Vinci SP robot usage for trauma cases in the English and German literature. While there are studies on urethral repair [17,18], no reports on severe traumatic situations were found. Furthermore, the only available report on the feasibility of robotics use for fracture treatment was based on a cadaveric study [8], aside from a single report for acetabular osteosynthesis [16]. Based on the available reports on da Vinci Xi robotics from the limited literature and our experience with both robots (da Vinci Xi and da Vinci SP), we decided to perform urethra repair using the da Vinci SP robot.

### 3.2. Intervention

**Figure 2:**
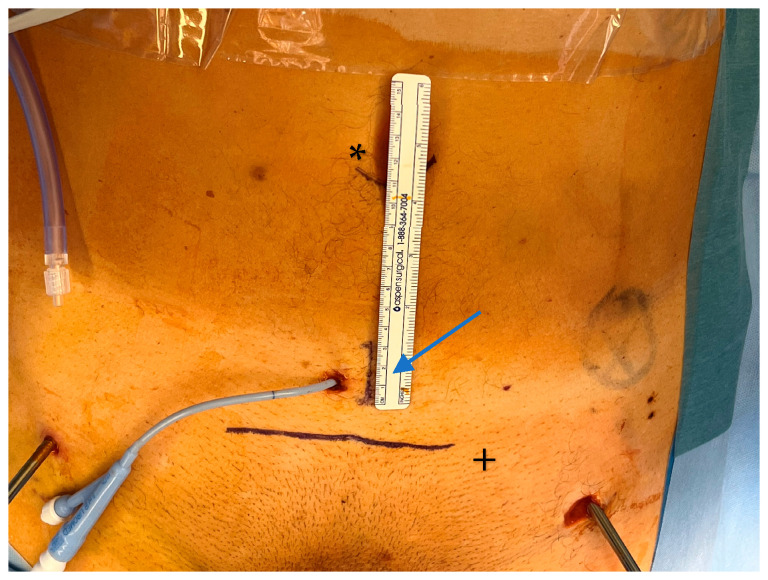
Patient was placed in the supine position with no Trendelenburg. A midline lower abdominal extraperitoneal incision (~2.5 cm; Figure 2—blue arrow/ruler), about 7 cm caudal of the navel (*), was performed to install the SP robot. The upper border of the anterior pelvis is marked (+). Suprapubic catheter and Schanz Pins from initial treatment remained in situ.

The patient was informed about the innovative procedure and the possibility of intraoperative conversion to established open pelvic surgery, with the primary goal of the procedure being to utilize the advantages of the SP robot for repairing the ruptured urethra. Accordingly, after the patient’s written consent was obtained, we used the robot to assist in the reconstruction of the anterior pelvic ring as follows: The patient was positioned on a carbon table to optimize potential intraoperative radiological imaging. A 2.5 cm single incision was performed next to the suprapubic catheter (Figure 2). Then, the carbon rods of the supraacetabular fixator were removed while Schanz pins remained in place as the pelvis was manipulated.

### 3.3. Approach

**Figure 3:**
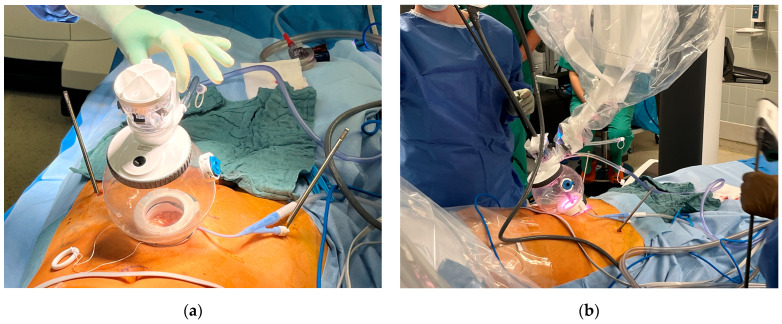
Mounting the da Vinci SP robot after removing carbon rods of the external fixator: (**a**) one single extraperitoneal approach is necessary to use the da Vinci SP robotic system; (**b**) up to four robotic instruments can be used via the single approach. Four instruments are inserted in this bubble-like connection.

The extraperitoneal approach to pelvic surgery using the da Vinci SP robotic system is a minimally invasive surgical technique primarily used in robot-assisted radical prostatectomy. This approach avoids entering the peritoneal cavity and is particularly useful in patients with prior abdominal surgery, or when avoiding bowel manipulation is advantageous. The patient is placed in the supine position with no Trendelenburg. Then, a midline lower abdominal incision (~2.5 cm; Figure 2—Blue arrow/ruler) is made. The anterior rectus fascia is opened, and the rectus muscle is split to enter the space of Retzius (extraperitoneal space). Blunt finger dissection is used to create the necessary space, and the da Vinci SP robotic system is then docked directly over the small access port (Figure 3). In our study, we further developed the space of Retzius under vision.

### 3.4. Urethral Repair

Due to the approximately 5 cm dehiscence of the urethra, endoscopic realignment would likely not have been successful. We therefore opted for robotic reconstruction. Due to the remaining dislocation of the anterior pelvic ring, visualization of the disrupted urethra was difficult. Nevertheless, the robot provided excellent visualization of the dislocated fragments. The bladder was opened ventrally over 2 cm, and trans vesical insertion of a guide wire was performed to mark the proximal end of the ruptured urethra, with the distal urethra marked using a Folley catheter. However, the initial compression of the anterior pelvic ring and posterior fixation tremendously impeded visualization of the ruptured urethra.

**Figure 4:**
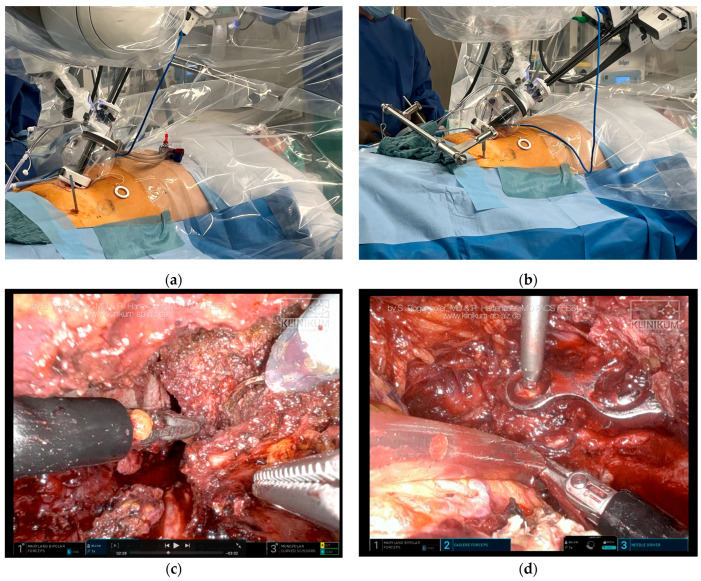
Visualization of ruptured urethra was limited due to the dislocated and compressed anterior pelvic ring. (**a**) Manipulation of the pelvis was performed by taking advantage of the Schanz pins from the initial fixator treatment. By doing so, the ruptured urethra could be visualized and repaired. (**b**) Visually controlled distraction was possible using an AO distractor. After resection of entrapped soft tissue (**c**), reposition and plate placement was possible (**d**).

To solve this, an AO distractor was utilized to distract the pelvis using the Schanz pins. Distraction was performed step by step under intrapelvic optical robotic control (Figure 4).

Because initial emergency stabilization of the pelvis was already performed and the posterior pelvic ring was rigidly fixed, relevant force was necessary to distract the compressed and comminuted anterior pelvic ring. After distracting the anterior pelvic ring via the AO distractor, the ruptured urethra was identified, and robotic repair was possible, subsequently being performed accordingly.

### 3.5. Fracture Repositioning and Osteosynthesis

The direct reposition of fragments using the da Vinci SP robot was not possible. However, entrapped soft tissue could be removed by taking advantage of the movement and visualization options of the robot. This essential step for adequate repositioning was performed without relevant blood loss. Repositioning itself was then performed using two additional 3 cm incisions on top of the anterior pelvic ring. Several reposition maneuvers using pelvic instruments from open surgery were necessary to ensure adequate positioning of the comminuted fracture. During this step, the robot could not perform adequate fragment manipulation. However, during the entire procedure, open visualization was not necessary. The optical possibilities of the robot allowed for visual control of all repositioning maneuvers; thus, the entire procedure remained minimally invasive.

After performing two additional incisions, there was a standard loss of gas, which did not obstruct the robotic surgery underway. The entire repositioning maneuver was performed without the need for radiological control, and the visualization and control of the intrapelvic fracture reduction was excellent. Additionally, trajectory estimation was possible due to optic control of the internal bony pelvis. Unfortunately, usage of the mounted robot and X-ray machine at the same time was not possible due to limited space. In order to use the X-ray machine, the robot had to be unmounted.

After reposition and temporary percutaneous K-Wire fixation, a 6-hole symphysis plate (PRO Pelvis and Acetabulum System, Stryker, Kalamazoo, MI, USA) was placed by the robot. The plate was inserted via the additional incisions without any fixation. Using the robot, the plate was then grabbed and placed on the anterior pelvic ring.

Orientation of the midline was challenging due to severe comminution issues. However, when the symphysis was intact, detection of the midline was easily possible with the robot. Moreover, the robot was able to hold the plate in the right place.

**Figure 5:**
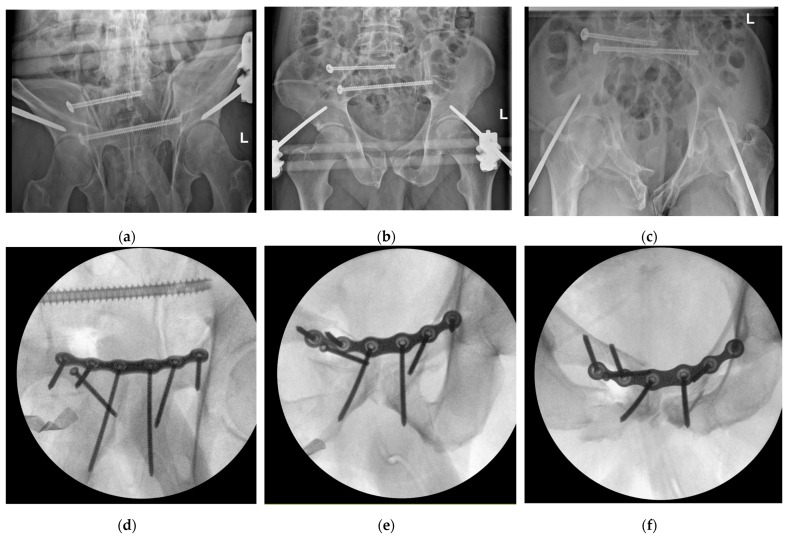
Pre- and intraoperative X-ray imaging documenting the remaining dislocation of the anterior pelvic ring: (**a**) outlet view after initial reconstruction of the posterior pelvic ring and fixator placement; (**b**) AP view after initial reconstruction of the posterior pelvic ring and fixator placement; (**c**) inlet view after initial reconstruction of the posterior pelvic ring and fixator placement; (**d**) outlet view of intraoperative imaging after repositioning and osteosynthesis; (**e**) AP view of intraoperative imaging after repositioning and osteosynthesis; and (**f**) inlet view of intraoperative imaging after repositioning and osteosynthesis.

Screw insertion using the robot portal was not possible due to undersized pelvic instruments and the need for trajectory optimization. However, it was possible to use the additional incisions for screw insertion under robotic control. Again, no open visualization was necessary. After complete fixation of the plate, the robot was dismounted, and X-ray control was performed. Two out of six screws needed to be optimized in terms of the need for a radiologically guided trajectory in the pelvis. No correction of the plate was necessary, and the radiological control of the reposition maneuver was judged to be satisfactory (Figure 5).

### 3.6. Outcomes

**Figure 6:**
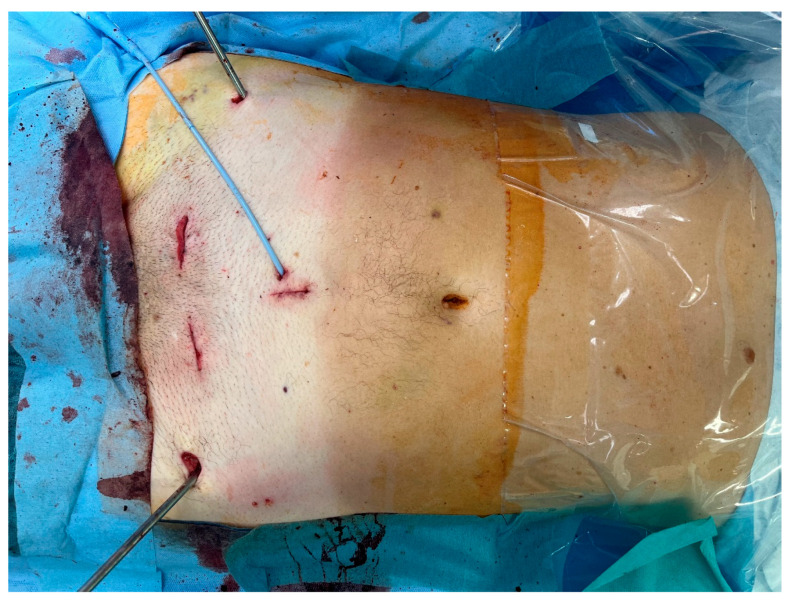
Complex urethral and pelvic fracture reconstruction was performed using 3 small incisions. Despite the need of 2 additional incisions for pelvic repositioning, this approach is still minimally invasive compared to traditional open pelvic trauma surgery.

The operation time was 6 h and 20 min, and the documented blood loss measured less than 250 mL. No transfusion was needed during surgery, nor was it needed post operatively. Additionally, there was no need for postoperative intensive care or observation, and no neurological deficits were obvious. Complex urethral and pelvic fracture reconstruction was performed using three small incisions (Figure 6), and a transurethral catheter was kept in place for 4 weeks to splint the urethra and to secure reconstruction. Additional suprapubic drainage remained in place to protect the reconstructed pelvis.

Mobilization began with full weight bearing on the left side and partial weight bearing on the right side. To secure the plate osteosynthesis in this highly comminuted situation, the supraacetabular external fixator was re-mounted and removed 4 weeks after surgery. After removal, full weight bearing was allowed. At the 6-week follow-up point, no secondary dislocation was detected in standard X-rays. This would not have been necessary in a less comminuted situation. Additionally, no secondary dislocation was observed. The patient was mobilized full weightbearing without pain. No motion restriction or limitation was obvious.

## 4. Discussion

### 4.1. Literature Review

We started our review according to the PRISMA guidelines. However, focusing on da Vinci robot usage in pelvic trauma surgery, only two reports have been found [8,16]. Furthermore, we have not been able to detect any report on da Vinci SP robot usage for trauma cases in the English and German literature. Due to this fact, we decided not to rely on the PRISMA recommendations. Consecutively, we have extended our literature search based on urologic robotic experience in our institution. While there are studies on urethral repair [17,18], there was an option to take advantage of a proven urologic procedure. In combination with the results from our initial review, we offered our patient the option to extend established urologic robotic surgery to fracture treatment using the da Vinci SP robot.

### 4.2. Interdisciplinary Approach

The approach to lower urinary tract reconstruction traditionally involves open pelvic surgery, but select cases may be suitable for robotic reconstruction. The robotic approach to lower urinary tract reconstruction is relatively novel, and preliminary data suggest that this approach may offer comparable rates of success, with less surgical trauma compared to open-surgery techniques [19]. Select case reports of robotic transperitoneal, multi-port urethroplasty with complete iatrogenic urethral transection after laparoscopic abdominal perineal resection [17] report early robotic repair to be feasible, with successful short-term outcomes. In our case, however, due to the approximately 5 cm dehiscence of the urethra, endoscopic realignment would likely not have been successful.

In general, multi-port robots use a transperitoneal approach, whereas single-port robots remain located in the extraperitoneal space, with this approach reportedly presenting advantages in urology [20]. The use of robotics in pelvic surgery facilitates complex urethral reconstruction in a minimally invasive fashion, with some reviews describing high success rates of urethral repair when using the robotic approach, comparable to those of open surgery, albeit with the advantage of faster recovery [21]. Most of these reports, however, are restricted to iatrogenic urethral injuries.

As we had no orthopedic robotic experience since, but an extensive background in traditional pelvic trauma treatment, an interdisciplinary discussion was held regarding the treatment options for our patient, in order to weigh their possible risks, burdens, and benefits. Due to the combined nature of the patient’s injuries, we decided to merge our knowledge in an interdisciplinary approach and utilize our multidisciplinary skills.

The combination of knowledge from different fields of surgery was extremely helpful during the whole process of complex treatment. Seeking advice, even beyond the own specialty, may be the first step to establish a standardized multidisciplinary collaboration in complex trauma cases. A short video (S1) is provided as Supplementary Materials.

Performing urethral visualization and repair required highly advanced techniques due to the patient’s compressed and comminuted anterior pelvic ring. This was possible by performing treatment as an interdisciplinary team. Moreover, after distracting the anterior pelvic ring, urethral robotic repair was possible.

The limited reports on pelvic trauma primarily concern multi-port robot use, in contrast to the single-port (da Vinci SP) system used in our system, which provided us with extraperitoneal access [8,16]. As our robotic center allows for the use of both robotic systems for routine surgery in urology and visceral surgery, we were able to take advantage of the refined, single-port robotic treatment option for urological surgery which has been reported for iatrogenic urethral injuries [17,21]. Moreover, by choosing the SP robot for urethral repair, we were able to investigate the potential of the robot in pelvic fracture treatment as a combined interdisciplinary procedure.

### 4.3. Fracture Treatment

The standard treatment for severely displaced pelvic fractures is open surgery. However, the invasivity and bleeding complications as a result of this method in particular result in longer ICU and hospital stays, longer recovery periods, worse outcomes, and higher mortality rates [22]. This might be reduced by using robots in surgery. In our case, despite the severe pelvic comminution, no blood transfusion and no ICU admission was necessary. Additionally, prolonged recovery did not occur.

Initial reports indicated that endoscopic pelvic fracture treatment was an available option [23,24,25]. Deemed the “total endoscopic anterior pelvic approach (TAPA)”, this method was published as a means for plate fixation of a symphyseal disruption [23]. Despite the total endoscopic technique, plate positioning and fixation was reported to be achieved through two additional, minimally invasive incisions. Additionally, the authors reported several advantages of this technique, such as better visualization, no detachment of the rectus abdominis muscle, and smaller skin incisions. Nevertheless, despite several attempts to minimize surgical approaches, such as the use of an internal fixator or percutaneous screw fixation, limitations remain. Internal fixator application of the anterior pelvic ring, termed INFIX, is also reported to be feasible [26]. However, the reposition quality remains inferior to that of open surgery [27]. Percutaneous screw fixation is another option for minimally invasive osteosynthesis of the anterior pelvic ring, with its biomechanical stability shown to be comparable to that of plate fixation [28,29]. Despite biomechanically comparable results and clinically proven advantages [30], a significant limitation remains concerning dislocated or comminuted fractures. Thus, plate osteosynthesis remains the treatment of choice in complex fracture patterns. In this context, initial reports on minimally invasive plate osteosynthesis techniques are available; however, the potential advantages from minimally invasive surgery are again limited in complex fracture patterns [31].

Taking all this into account, the endoscopic and minimally invasive advantages of robotic systems may be augmented with precise preparation skills, which would lead to reduced bleeding and soft tissue damage.

The general feasibility of robotics was demonstrated with the use of a da Vinci Xi robot in a cadaveric setup [8], with robot-assisted, endoscopic plate osteosynthesis performed on the symphysis and anterior acetabular column. Additionally, robot-assisted plate osteosynthesis of the anterior pelvic ring and acetabulum was reported to be feasible with the Vinci Xi systems, with other advantages of robot-assisted procedures being the wide range of robotic arm movement, magnification of 3D visualization, and highly accurate preparation of the situs [8]. Kabir et al. took advantage of these benefits by using this technique for robot-assisted acetabular repair and osteosynthesis using the da Vinci Xi robot, stating that robot-assisted plate fixation is a promising alternative for bony injuries of the anterior pelvic ring. Other advantages are reported to be optimal visualization in combination with minimal incisions [16]. Despite these reports of usability and first clinical experiences, however, the use of robots in the reduction of dislocated fractures remains uncertain, and no reports on severely displaced anterior pelvic ring fractures are available.

Based on the procedure described, we are able to confirm the advantages reported in the clinical report on acetabular surgery. Furthermore, we report excellent visualization of fractures, urethral repair, and visualization during repositioning maneuvers. Additionally, we were able to perform resection of entrapped tissue in a minimally invasive and highly controlled method.

### 4.4. Limitations and Need for Development

While robotic urethral repair was possible via the single-port robotic approach only, fracture reposition, and screw placement required two additional incisions. This necessary increase in invasivity should be the focus of robotic refinement in this field. By developing specific instruments for complete single-port repositioning and osteosynthesis, the advantages of this approach will increase. Thus far, only robot-assisted treatment is possible; full robotic treatment requires profound development in this field.

Nevertheless, no open visualization was necessary in order to place the screws. Reposition was difficult due to the already fixed posterior pelvic ring and the need to apply relevant force for the reposition maneuvers. Ultimately, the combination of an external AO distractor using the supraacetabular Schanz screws from the initial emergency stabilization procedure and conventional techniques from open surgery resulted in acceptable reposition results.

As reported by others [8,16,23], an additional incision is necessary to facilitate reposition and to place screws, while the plate can be perfectly positioned and held in place by the robot. This was also the case with the single-port robot. Nevertheless, the SP robot only requires one incision for entry, indicating that the overall need for incisions can be minimized. However, this potential advantage is contradicted by the need for additional incisions for reposition and screw placement purposes later on.

As no specific repositioning and osteosynthesis tools are available for da Vinci robot systems, there are limited options to manipulate and fix bone. Additionally, instrument development is necessary for broader adoption, extended treatment options, and the interdisciplinary use of SP robotics.

The development of special robot-based instruments may potentially solve this issue and may even allow for fragment manipulation and reposition via single-port robots in the future. To accomplish this, the robot must be adapted to apply high levels of force. As of the present, the focus of the da Vinci robot is sophisticated soft tissue surgery, which does not require the application of increased force. Far from it, high levels of force must be prevented in order to avoid damaging soft tissue during surgery. This must be addressed once this system is to be used for fracture manipulation and reposition, given that high levels of force are necessary to achieve anatomic reduction in pelvic fractures. As the da Vinci robot was not able to be used for repositioning maneuvers, using the robot for soft tissue dissection, fragment cleaning, and superior control of minimally invasive reposition instead proved to be highly beneficial.

We used a 6-hole symphysis plate during the procedure. Considering the highly comminuted situation, a longer plate would have potentially led to improved anchorage and improved stability. However, contouring the plate and fixation around the acetabulum would have been even more demanding. Despite the reports on acetabular robotic plate fixation [16], we decided to combine our internal osteosynthesis with the already placed supraacetabular external fixator for 4 weeks, which would not have been necessary in a less comminuted situation. After removal of the external fixator, no secondary dislocation was observed.

Similarly to the patients in other reports on robotic surgery, our patient rapidly recovered. No postoperative intensive care was necessary despite the invasive procedure and prolonged operation time. This indicates that, despite the limitations presented, this technique is promising and can be refined to both improve quality and reduce invasiveness. However, there is verry little knowledge in the literature so far. In this context, further clinical experience and technical developments are required before broader adoption.

Unlike urology, where robotic surgery is already established, its use in orthopedics is still debatable. In a recent review on robotics in orthopedics, Hu et al. concluded that robot-assisted surgery was promising but required “addressing the delineated challenges, pursuing with optimism, while critically and meticulously ensuring thoughtful execution, outcomes, and cautious cost-effective integration into clinical practice” [14].

This report could contribute to the development of this promising technique. Given that our patient benefited from our interdisciplinary cooperation during the indication, planning, and execution stages of this study, we can conclude that the robot-assisted surgery performed is a key effort directed towards improving surgical care for our patients.

## 5. Conclusions

To our knowledge, this is the first report on the clinical use of the da Vinci SP robot for combined urological and orthopedic trauma care of the pelvis, with results indicating the successful urethral repair of pelvic ring disruption as a result of severe trauma. Additionally, successful robot-assisted treatment of a severe pelvic fracture was performed, indicating that this report on acute treatment is the first of its kind. Single-port da Vinci SP robots are able to assist in complex pelvic fracture reconstruction, given the known advantages of urologic robotic surgery, such as improved intrapelvic preparation and visualization, especially in cases of additional urethral injury. Interdisciplinary cooperation is necessary in order to take advantage of this promising technique. In established robotic centers in particular, the potential to refine surgery beyond the horizon of the immediate field is considerable. However, for a complete robotic fracture procedure, special instrument development for reposition and screw placement is necessary.

## Data Availability

The data presented in this study may be available on request from the corresponding author under consideration of patient specific data protection restrictions.

## Supplementary Materials

The following supporting information can be found in the dropbox: https://doi.org/10.5281/zenodo.16931155 (accessed on 27 September 2025), Video S1: Summary of robot-assisted pelvic reconstruction and osteosynthesis.
